# Novel PET tracers to distinguish the nature of residual masses after the completion of chemotherapy in metastatic testicular germ cell tumours: A systematic review

**DOI:** 10.1007/s00259-026-08044-5

**Published:** 2026-07-02

**Authors:** Alessandro Bertocchi, Fabio De Vincenzo, Matteo Perrino, Nadia Cordua, Priscilla Guglielmo, Marta Aliprandi, Luigi Giovanni Cecchi, Antonio Federico, Antonella Panzardi, Armando Santoro, Laura Evangelista, Paolo Andrea Zucali

**Affiliations:** 1 Department of Biomedical Sciences, Humanitas University, via Rita Levi Montalcini 4, 20072 Pieve Emanuele, Milan, Italy; 2 Medical Oncology and Haematology Unit, IRCCS Humanitas Research Hospital, via Manzoni 56, 20089 Rozzano, Milan, Italy; 3 Nuclear Medicine Unit, Humanitas Gavazzeni, Via Mauro Gavazzeni 21, 24125 Bergamo, Italy; 4 Nuclear Medicine Unit, IRCCS Humanitas Research Hospital, via Manzoni 56, 20089 Rozzano, Milan, Italy

**Keywords:** Testicular germ cell tumours, Residual masses, PET tracer, PET scan

## Abstract

**Background:**

Testicular germ cell tumours (TGCTs) are the most common malignancy in young adult males. After chemotherapy, the evaluation of residual masses is critical. However, the current standard imaging modality, [^18^F]-FDG PET, has limited specificity—particularly in distinguishing viable tumour from necrosis or mature teratoma. This limitation often leads to unnecessary surgery or misinterpretation of treatment response.

**Objectives:**

This systematic review aims to explore and evaluate novel PET tracers that could improve diagnostic accuracy in TGCTs, especially in post-chemotherapy settings.

**Methods:**

A comprehensive literature search was conducted on PubMed and Scopus through December 2025, using terms including “testicular,” “germ cell tumour,” “PET scan,” and “novel.” Studies focusing solely on [^18^F]-FDG were excluded. Preclinical studies, case reports, and clinical trials investigating alternative PET tracers or relevant molecular targets (e.g., ghrelin, claudin-6) were included. The quality of selected articles was assessed by using CASP for the qualitative research.

**Results:**

A total of 17 studies met the inclusion criteria. Emerging PET tracers evaluated include [^18^F]-FLT, FAPI, αvβ3-integrin-targeted agents, [^18^F]-fluciclovine, PSMA-targeted tracers, and those based on ghrelin and claudin-6. Among these, αvβ3-targeted imaging has shown promising preclinical results in differentiating mature teratoma from necrosis. Ghrelin receptor tracers appear promising due to their high expression in TGCTs, although they remain in preclinical development. Claudin-6, a tight-junction protein, is highly expressed in most TGCT histologies and may be a viable molecular target, as supported by early-phase clinical trials.

**Conclusions:**

Novel PET tracers represent a promising avenue to refine TGCT management. αvβ3-integrin and ghrelin-based tracers are particularly noteworthy for their potential to guide decision-making after chemotherapy. However, further clinical validation is required before routine implementation. These innovations could significantly reduce overtreatment and enhance personalized care in TGCT patients.

**Supplementary Information:**

The online version contains supplementary material available at https://doi.org/10.1007/s00259-026-08044-5.

## Introduction

Testicular cancer (TC) is a rare disease, predominantly affecting young males between 15 and 40 years old among whom it is the most common solid tumour. It represents 1% of male neoplasms, but its incidence is increasing. In 2020 alone, 74,500 new cases, a third of which in Europe, and nearly 9500 deaths were registered worldwide [1, 2]. The predominant histology of TC is represented by Germ Cell Tumours (GCTs) among which seminoma and non-seminoma are the more frequently diagnosed histotypes (> 95%) with the first one accounting for half of the cases [3]. Non-seminomatous GCTs (NSGSTs) include embryonal carcinoma, teratoma, yolk sac tumour, choriocarcinoma and mixed forms.

Ninety-five percent of GCTs originate from testis, occasionally in extragonadal primary sites [4]. Diagnosis requires testicular ultrasound, the dosage of serum tumour markers beta-human chorionic gonadotropin (β-hCG), alpha-fetoprotein (AFP) and lactate-dehydrogenase (LDH) and systemic imaging [4]. A correct initial staging is crucial to set up a correct therapeutic strategy because to each stage correspond a specific treatment, both for SGSTs and NSGSTs [5]. Classification of metastatic gonadal and extragonadal GCT is based on TNM, clinical stage according to the Union for International Cancer Control (UICC), and the International Germ Cell Cancer Collaborative Group (IGCCG) prognosis group [4]. CT scans of thorax, abdomen and pelvis are recommended for TC staging. However, CT scans may not detect normal sized pathologic nodes. Magnetic resonance imaging (MRI) of the abdomen may be used for staging in case of allergy to iodine-based contrast media or in the aim to reduce radiation exposure, especially during follow-up, with similar accuracy to CT scan in the detection of retroperitoneal nodal enlargement. The main objections to its routine use are its high cost and limited availability. MRI should be preferentially used in the evaluation of cerebral or spine metastases as well as bone scan in case of suspected bone metastases [4]. There is no evidence to recommend the use of Fluorodeoxyglucose-positron emission tomography ([^18^F]-FDG PET) for TC staging. However, [^18^F]-FDG PET is recommended, after at least 2 moths from the last cycle of chemotherapy, because imaging acquired at earlier time points may be influenced by treatment-related inflammation, for patients with residual seminoma masses > 3 cm and normal levels of markers post-chemotherapy, to assess residual viable tumour [4], because of its high predictive negative value (> 90%) [6]. After chemotherapy of stages II-III seminomas, a residual mass could be found in more than 50% of cases [7, 8] and, if the diameter is ≥ 3 cm, the probability of discovering vital lesion varies, according to various studies, from 11% to 37% [9, 10]. If the residual lesion is < 3 cm, with the normalization of the values of LDH, β-HCG and AFP, the use of [^18^F]-FDG PET is not recommended and patient is commonly candidate for follow up [4]. When a post-chemotherapy mass remains positive at reclassification with [^18^F]-FDG PET with no volume increase, a follow up [^18^F]-FDG PET should be performed six weeks later. Unfortunately, up to 75% of PET scans could be falsely positive [6] but 11%−37% of seminomatous residual tumour masses > 3 cm could contain viable tumour [9, 10] so, before further treatments, a biopsy should be considered [4, 5].

On the other hand, [^18^F]-FDG PET is not recommended in the re-staging of patients with NSGCT after chemotherapy considering the low sensitivity due to the risk of presence of teratoma features [11]. In fact, [^18^F]-FDG-PET has the significant limitation of not being able to distinguish teratomatous lesions [12] and in cases of NSGCTs this could lead to false negative exams. Unfortunately, after the completion of the systemic treatment of a stage II-III non-seminoma, the probability of presenting a residual mass ranges from 30 to 70%, even if the concentration of blood markers is normal [13]. Those lesions are characterized by necrosis in 40% of cases, mature teratoma in 50% of times, and vital non-seminomatous cancer cells in 10% [14–18]. In addition, a few small retrospective studies have investigated whether [^18^F]-FDG-PET may have a prognostic role in this malignancy, although without positive results [19, 20]. Unlike other malignancies, according to international guidelines, prognostic assessment in patients with testicular cancer should be based on clinical staging systems only [4]. Mature teratoma is particularly resistant to chemotherapy and it is cured only by surgery [21]. If the residual mass is > 1 cm, it should be resected by surgery and, if fibrosis or mature teratoma is found, the patient does not require further treatments. In case of retroperitoneal masses, nerve sparing-RPLND is the preferred technique, but it is a difficult procedure, heavily dependent on the surgeon experience and with potential severe adverse effects, such as retrograde ejaculation [22]. Therefore, a high number of patients with complete responses to chemotherapy are treated with major surgery without benefit and it is easily knowledgeable that a radiological method capable to distinguish between a fibrotic and a vital lesion could have an extremely important impact on the current clinical practice in the treatment of advanced non-seminoma.

The use of a novel PET tracer, capable of reliably distinguishing testicular germ cell lesions, could potentially aid in determining whether the potential residual disease still possesses vital components, particularly in cases of non-seminomatous tumours, and the presence of teratomatous elements. The aim of this paper is to describe and evaluate new potential PET tracers capable of achieving this role.

## Materials and Methods

Comprehensive research of the literature was conducted using PubMed and Scopus databases until December 2025 employing the database specific syntax and using a combination of the words “testicular”, “germ cell tumour”, “PET scan”, “novel” with boolerian words. Furthermore, we included additional terms, such as “ghrelin” and “claudin 6”, due to their hyperexpression in TGCTs, also in the absence of dedicated PET imaging method. Given that [^18^F]-FDG PET scan is already in use in clinical practice and the target of this review is to explore the use of novel PET tracers with possible future use, we decided to exclude papers concerning its application and research about this topic. Additional references have been attached to provide context, found from manual research in the databases and from key words from other articles.

The identified papers have been selected using the PRISMA flowchart. Since the scarcity of evidence in this context, a discussion about potential target and PET tracers useful in this context, that the authors considered promising, was reported.

We included pre-clinical studies, case reports and clinical trials. The studies included in this review have been evaluated using the CASP checklist for qualitative research v2024.

## Development of novel PET tracers for germ cell neoplasms

**Fig. 1:**
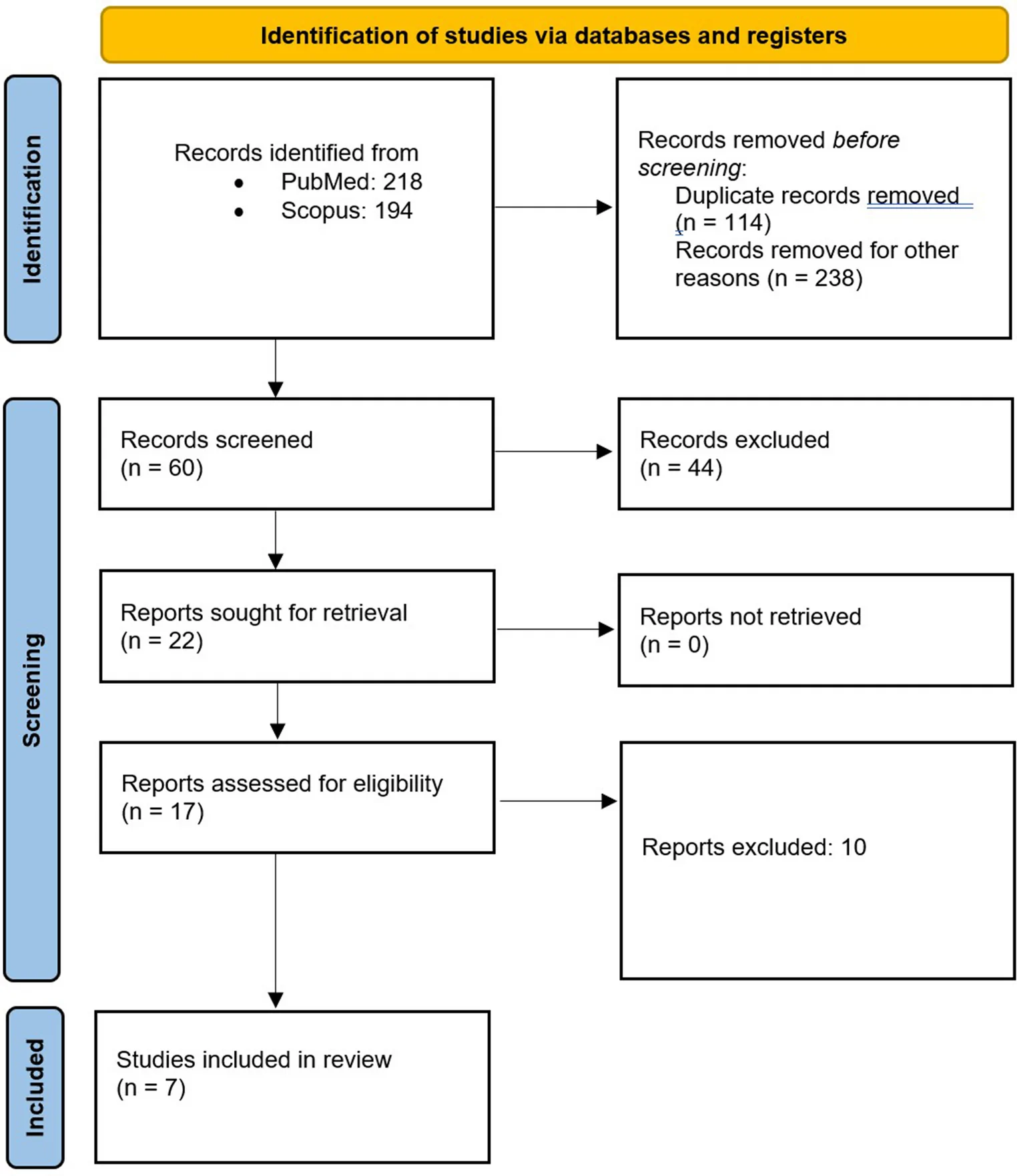
Prisma flow chart

After the screening a total of 17 papers have been included in our review, demonstrating the great possibility for future research. The PRISMA flow chart in Fig. 1 summarizes the process we followed.

To date, there are many PET imaging agents in development in this patient setting. During our research we found scientific papers about tracers based on FLT, αvβ3, [^18^F]-fluciclovine, FAPI and PSMA in this context and evidence of a possible use of ghrelin and claudin 6 as PET tracers for germ cell tumours.

### Radioimmunodetection of GCT antigens

In the 1980 s, conventional imaging was not as developed and reliable as it is today; therefore, radioimmunoscintigraphy was explored for the diagnosis of TCs by targeting antigens typically expressed by these neoplasms. The principle of this technique was based on the accumulation of antibodies labeled with gamma-emitting radionuclides on the tumour surface, followed by image acquisition using a gamma camera. In 1981, Javadpour et al. published a small study involving five patients who, during the diagnostic work-up, received ^131^I–labeled antibodies against HCG or AFP, followed by total-body photoscanning [23]. In 1985, Epenetos et al. published a trial in which 15 patients with testicular, ovarian, or cervical cancer received an ^111^In–labeled antibody directed against placental alkaline phosphatase (PLAP). In this latter study, the technique demonstrated good reliability in patients with active disease and, in one patient with teratoma, enabled the detection of a nodal teratoma metastasis that was not visible on conventional imaging [24]. The progressive development of conventional imaging techniques, together with the limitations of radioimmunodetection, including the potential immunogenicity of antibodies and the low spatial resolution for small lesions [25], ultimately led to the abandonment of this approach. Although these agents were not strictly PET tracers, we decided to include this technique in our review because of the historical relevance of these early experiences.

### Thymidine analog 39-deoxy-39-[^18^F]-fluorothymidine (FLT) PET scan

In 2011 a group of researchers in Germany evaluated the use of FLT-PET scan to differentiate the content of residual masses after chemotherapy in metastatic germ cell cancer [26].

FLT is a cell proliferation marker. The compound after entering the cell is trapped by the phosphorylation by thymidine kinase 1 and, since this enzyme is closely associated with cell proliferation being a key enzyme in the salvage pathway of DNA synthesis, the accumulation of [^18^F]-FLT depends on the expression of thymidine kinase 1 and hence to cell mitosis [27]. This characteristic makes [^18^F]-FLT a reliable proliferation-tracer to use in PET scans and, in fact, has been proven able to identify fast proliferating tissue in a variety of tumours like lung cancer and colorectal cancer [28, 29].

The abovementioned group from the University and Hospital of Tuebingen compared the performance of [^18^F]-FLT and [^18^F]-FDG PET scan in eleven patients with metastatic germ cell tumours before chemotherapy, after the first cycle (early response) and 3 weeks after completion of the treatment. They compared metabolic results with contrast-enhanced CT findings, tumour marker levels and validating them with histopathologic examination of resected residual masses (7 patients) or clinical and radiologic follow-up for at least 6 months (4 patients).

Ten out of 11 patients were diagnosed with metastatic non-seminoma and 1 with metastatic seminoma, all of them with gonadal primary. According to the classification of the International Germ Cell Cancer Collaborative Group, 6 patients were poor-prognosis GCT, 4 intermediate-prognosis GCT, and 1 good-prognosis GCT. A subject was defined as responder if showing a complete radiological remission, necrosis at histological examination residual masses or a marker-negative partial remission within a minimum progression-free interval of 6 months.

Totally, 33 [^18^F]-FDG PET scans and 31 with [^18^F]-FLT PET scans were performed. A single patient showed no [^18^F]-FLT tumour uptake on the pre-treatment scan; therefore no subsequent [^18^F]-FLT examination was performed. At the end of the treatment, 6 out of 11 men responded to chemotherapy.

The pre-treatment analysis of all 11 patients showed an increased uptake of [^18^F]-FDG (SUV mean 8.8, 2.9–15.0) and everybody except 1 patient had a tumour uptake of [^18^F]-FLT but with a more moderate signal (mean SUV 3.7, range 1.7–9.7). Cancerous lesions showed a heterogeneous uptake without any visible correlation between the two tracers. Early response, after 1 cycle of chemotherapy, and end-of-treatment monitoring showed a reduction of both [^18^F]-FDG and [^18^F]-FLT uptake in the reference lesions in all 11 patients, but results were inconsistent. In 2 patients teratoma and in other 2 vital tumour was histologically found plus 1 further man early relapsed after the completion of the chemotherapy. In all of those 5 subjects [^18^F]-FLT PET scan failed to show an uptake of the residual mass and [^18^F]-FDG succeeded to show a vital lesion with vital tumour only in 1 man. Considering the 6 patients who responded to therapy, both [^18^F]-FLT and [^18^F]-FDG were truly negative.

After 1 cycle of chemotherapy, sensitivity, specificity, positive predictive value and negative predictive value for detection of a vital lesion were 60%, 33%, 43%, and 50%, respectively, for [^18^F]-FDG and 60%, 80%, 75%, and 67%, respectively, for [^18^F]-FLT. The respective values after the end of chemotherapy were 20%, 100%, 100%, and 60% for [^18^F]-FDG and 0%, 100%, 0%, and 50% for [^18^F]-FLT.

Considering the initial question of using a PET tracer to reliably identify vital tumour of teratoma in residual masses after the completion of chemotherapy in GCT, this trial demonstrated that [^18^F]-FDG PET scan was characterized by a low negative predictive value and [^18^F]-FLT PET scan completely failed to recognize vital lesions [26].

### αvβ3 imaging

After the publication of abovementioned article about [^18^F]-FLT on the Journal of Nuclear Medicine, the same editor in May 2011 published a letter sent by a French group from the Nuclear Medicine department of the Centre Francois Baclesse of Caen about a successful attempt in identifying vital teratoma from necrosis in residual non seminomatous GTC masses using imaging of αvβ3 integrin in a preclinical setting [30].

Integrins are the main adhesive cell adhesion receptors for components of the extracellular matrix, 24 different proteins originated from the combination of 18 α and 8 β subunits. Integrins are not simple anchor points but transmembrane receptors which can bind different ligands, capable of turning on various intracellular pathways and regulating various cellular functions. Integrins are crucial to cancer cells being able, by linking to various ligands, to induce drug resistance, influence tumour–stroma crosstalk, support the formation and regulate the formation of invasive protrusions, white blood cell trafficking and activation, chronic inflammation, immune mimicry and angiogenesis [31].

αvβ3, also known as a receptor for vitronectin, a glycoprotein of the hemopexin family which promotes cell adhesion and spreading, binds to a wide variety of molecules with an Arg-Gly-Asp triple-peptide motif (RGD) like fibronectin, fibrinogen, von Willebrand factor and the previously cited vitronectin. αvβ3 integrin is implicated in various process but, most importantly, angiogenesis. It elicits this function by binding fibroblast growth factor-2 (FGF2), metalloproteinase MMP-2, activated PDGF, insulin, and VEGF receptors. Its role in angiogenesis is supported by preclinical data about the induction of endothelial cell apoptosis and, therefore, angiogenesis inhibition, after the pharmacological blocking of this integrin. It is now comprehensible how the expression of this integrin can be considered as a marker of neoangiogenesis, being highly expressed in vessel-rich tissues and, therefore, in cancer tissue [32–35].

The French group studied if a PET scan with a technetium-marked tracer carrying the Arg-Gly-Asp domain, capable of binding αvβ3, could distinguish mature teratoma from necrotic lesions in a preclinical model ([^99m^Tc]HYNIC-RGD [6-hydrazinonicotinic acid conjugated to cyclo(Arg-Gly-Asp-D-Phe-Lys)).

After cultivating two human embryonal carcinoma (EC) cell lines, NTERA-2 because of its pluripotency and NCCIT to exploit its sensitivity to cisplatin, the researchers grafted 20 specimens of 4 weeks old nude rats with six tumours each in the same spots, when the largest tumour reached 7–10 mm diameter the experiment began [36, 37].

Mature teratoma model was obtained by exposing NTERA-2 xenografts to cisplatin + all-trans retinoic acid (ATRA) (group 1) while necrosis by exposing NCCIT derived grafted animals to cisplatin and etoposide (group 3); another group of rats were kept as control (group 2). Models were then divided into two tests, 2 small groups of NTERA-2 grafted rats. Group 1 and 2 (group 1 and 2) were subjected to a preliminary experiment to test whether mature teratoma model would exhibit a [^18^F]-FDG pattern like that of a mature teratoma found in real life NSGCT patients following chemotherapy and the impact of the differentiation process on [^18^F]-FDG uptake. A second, larger, group of rats (groups 3,4 and 5) were subjected to the main experiment to verify if a marked RGD-PET scan could distinguish mature teratoma from necrosis.

The chosen tracers for the study were [^99m^Tc]HYNIC-RGD [6-hydrazinonicotinic acid conjugated to cyclo(Arg-Gly-Asp-D-Phe-Lys)] and [^18^F]-FDG, administered by vein infusion to the specimens.

The preliminary experiment showed that in control grafted embryonal carcinoma, [^18^F]-FDG uptake was heterogeneous; while it was significantly reduced in NTERA-2 rats treated with cisplatin+ATRA (EC differentiated in mature teratoma) similarly as observed in human chemotherapy-induced mature teratomas, with a reduction after a 4-week period of SUVmean of 47% and SUVmax of 37% and 63% and 48%, respectively, after 8 weeks. The preliminary study succeeded to demonstrate that in this model, as in real clinical scenarios, [^18^F]-FDG uptake in mature teratomas is less avid than the one of the germinal tumours they derive from. Other than that, histological examination confirmed that cisplatin+ATRA had altered the growth of NTERA-2 EC and induced the differentiation in mature teratoma.

Regarding the main experiment, a total of 6 NTERA-2 grafted rats exposed to cisplatin+ATRA bearing a total of 36 mature teratomas were imaged, 1 of which (6 MT) was involved in a blocking experiment to verify if the chosen tracer effectively bound αvβ3. In this xenograft a large amount of non-marked RGD was injected and the subsequent PET scan with the marked-RGD showed no uptake, demonstrating that the compound effectively was binding the αvβ3 integrin. Regarding the remaining 5 rats with a total of 30 mature teratomas, a clear and visible uptake was observed in 24 tumours with the residual 6 teratomas being masked by surrounding areas of intense physiological clearance (bladder and colon excretion). All the lesions were histologically confirmed to have characteristics compatible with mature teratomas.

Two NCCIT grafted rats, previously exposed to etoposide+cisplatin to induce necrosis, with 12 necrotic lesions were also imaged and, in contrast to that observed in mature teratomas, the HYNIC-RGD uptake was very low. The tumour (T)/mediastinum (M) ratios of mature teratoma lesions ranged from 2.88 to 8.97 (mean ± SD 4.29 ± 1.57) while of necrotic lesions from 0.88 to 1.53 (mean ± SD 1.3 ± 0.26).

All the lesions were histologically studied and, other than confirming their nature of mature teratoma or necrosis, were subjected to immunohistochemistry using the monoclonal antibody LM609 directed against the αvβ3 heterodimer. All tumour samples of mature teratomas showed a strong staining on the cell membranes of glandular structures of the mature teratoma and were surrounded by many host-derived blood vessels. In contrast, necrotic lesions did not show any significant staining for αvβ3 integrin.

In conclusion, the study was successful in distinguishing between mature teratoma and necrosis in human embryonal-carcinoma-grafted preclinical models [38]. Although [^99m^Tc]HYNIC-RGD is a SPECT radiotracer rather than a PET tracer, we decided to include this study in our review because it explored a highly innovative molecular target, namely αvβ3 integrin expression, and the study design included a direct comparison with [^18^F]-FDG PET/CT.

Following these results, the same group conducted a single group phase I/II trial, TERMATEP or NCT02317393, from December 2014 to March 2020, recruiting 16 patients with metastatic non-seminoma with residual lesions after the completion of I line chemotherapy and for which the surgery is planned. The trial is evaluating as primary endpoint the proportion of teratoma, defined as efficacy to differentiate mature teratoma and necrosis, and the metabolic profile as secondary endpoint, defined as number of patients having at least one anatomopathological-confirmed tumour with a RGD uptake. Unfortunately, to date, no official results or publications are available.

### FAPI PET/CT

FAPI is an inhibitor of the fibroblast-activation-protein (FAP), which is expressed by cancer-related fibroblasts in the tumour microenvironment of solid malignancies. It is increasingly used for PET imaging, by employing diverse peptides, radiolabelled with ^68^Ga or ^18^F radioisotope. The pilot study by Kidess et al. evaluated the feasibility of [^18^F]-FAPI-74 PET/CT for imaging and staging of gonadal and extragonadal GCT. Five patients undergoing chemotherapy or residual tumour resection were examined, and tracer uptake was correlated with FAP expression in histopathology. The study demonstrated a positive correlation between PET tracer uptake (SUVmax) and FAP expression (*r* = 0.80), suggesting that FAPI PET/CT can provide molecular information about the tumor microenvironment. In some cases, increased uptake was observed in teratoma and fibrotic or inflammatory tissue, indicating potential false-positive findings, therefore requiring further studies for establishing its role in this subset of patients [39].

The diagnostic performance of the investigated PET tracers is summarized in Table 1. Where available, reported sensitivity and specificity.

**Table 1:** Sensibility and specificity of tracers (when available)

TRACER	TARGET	STUDY	*N*. OF SUBJECTS	SENSITIVITY	SPECIFICITY
Thymidine analog 39-deoxy-39-[ ^18^ F]-fluorothymidine (FLT)	Thymidine kinase 1	Pfannenberg et al., 2010 [26]	11	(after end of CT) 0%	(after end of CT) 100%
[ ^18^ F]-fluciclovine (Axumin)	Sodium-dependent amino acid transporters	TESTPET Woldu et al., 2021[40]	9	29%	33%
[ ^18^ F]F-FAPI-74	Fibroblast-activation protein (FAP)	Kidess et al., 2025[39]	5	(only 3 pts underwent RTR) 100%	(only 3 pts underwent RTR) 0%

### PSMA PET

Prostate Specific Membrane Antigen (PSMA) is a surface receptor antigen expressed in prostate tissue and tumour-associated neo-vasculature. It is a glutamate carboxypeptidase type II non-secreted transmembrane protein composed of 750 aminoacids and it is highly expressed by prostate cancer cells, becoming a crucial biomarker used for diagnosis and treatment-monitoring of this tumour. The biological role of this glycoprotein is to remove the g-linked glutamates from folate, providing deglutamated folate for absorption, in fact the official name of the gene coding for PSAM is folate hydrolase 1 (FOLH1) [41, 42].

Giving its frequent exposition on prostate cancer tissue and tumour-associated neo-vasculature, different PET tracers have been developed, like ^68^Ga-PSMA and [^18^F]-PSMA, with the ability to bind the PSMA on the surface of cancerous cells and provide an image through a PET scan [43].

Other than prostate cancer, the use of PSMA PET scan in other tumours is a highly active field of research and this method is being studied for example in mesothelioma, lung cancer, various gastrointestinal neoplasms and, especially, renal cell cancer but unfortunately there are no studies about the application of PSMA pet in testicular germ cell tumours [44, 45].

In May 2021 Clinical Nuclear Medicine published an abstract of a case report from a Turkish group about a 46-year-old patient with a metastatic mixed testicular mixed germ cell tumour and was previously administered standard therapies for the disease. A ^68^Ga-PSMA PET/CT was performed and demonstrated an intense uptake so, subsequently, the patient was given ^177^Lu-PSMA radionuclide therapy with a mixed response. After 1 month from the administration an increase in blood alpha-fetoprotein was found and, after a ^68^Ga-PSMA PET/CT, new PSMA-negative lesions were detected. Unfortunately, no detailed full text is available, and no more information can be found about that experience [46].

### [^18^F]-fluciclovine (Axumin)

A study evaluated the performance of [^18^F]-fluciclovine PET scan in detecting residual non-seminomatous germ cell tumour prior to retroperitoneal lymph node dissection [47]. Anti-1-amino-3-[^18^F]-flurocyclobutane-1-carboxylic Acid, commonly called [^18^F]-fluciclovine and sold with the commercial name Axumin, is a [^18^F]-synthetic analog of the amino acid L-leucine and has been proven capable of diagnosing prostate cancer recurrence. Axumin is an FDA-approved PET tracer for the detection of recurrent prostate cancer.

In this prospective phase II study (TESTPET), a total of 10 men, 9 of which received chemotherapy before, underwent a [^18^F]-fluciclovine PET scan prior being subjected to retroperitoneal l lymph node dissection (RPLND). Unfortunately, this procedure showed to have low sensitivity and specificity. Only 3 patients showed a correlation between PET uptake and histopathology with 2 true positive and 1 true negative, 5 patients were found to be false negatives and 2 false positives: sensitivity was 29% and specificity 33% [40].

### Ghrelin receptor

The ghrelin receptor is a member of the growth hormone secretagogue (GHS-R) family that contains two isoforms, such as GHS-R1a and GHS-R1b, with the latter being non active in human metabolism. GHS-R1a, when bound to human ghrelin, regulates tissue growth and differentiation but has even a crucial role in cancer proliferation, apoptosis, invasion and migration [48]. Giving its expression on different cancers tissue, there is a high interest in developing PET tracers using marked ghrelin [49–52].

A study investigated the expression of ghrelin and GHS-R1a in healthy testis tissue and testis-derived germ cell tumour using immunohistochemistry, revealing that the expression of ghrelin was present in Leydig and Sertoli cells, but not in germ cells, undifferentiated Leydig cell tumour and germ cell tumors [53]. Regarding GHS-R1a, the possible target for a radioactive-ghrelin PET tracer, it was found to be expressed with different intensities by non-neoplastic testis tissue but, most importantly, by all kind of testicular tumours like differentiated and poorly differentiated Leydig cell tumour (++), seminoma (++) and embryonal carcinoma (++) [52].

Giving the widespread expression of GHS-R1a in germ cell tumour, a reliable ghrelin PET tracer could represent a promising new method for the molecular imaging of those malignancies [54].

The development of a ghrelin-based PET tracer is an interesting topic. In fact, some tracers have already been developed and there is an active academic discussion regarding its possible clinical implications [55].

The interest in a ghrelin-based PET tracer does not only involve oncology, but also some applications in endocrinology, internal medicine and cardiology have been advised. It is known that ghrelin is produced in cardiomyocytes and, by binding to its receptor, promotes cardiomyocyte survival. Thus, the therapeutic use of ghrelin after myocardial infarction could be exploited to enhance the prognosis of cardiac ischemic stroke patients. A recent study showed that a [^18^F] tracer ([[^18^F]]LCE470, a quinazolinone derivative) has been developed and successfully utilized to investigate the in vivo and ex vivo expression of the ghrelin receptor in a canine model of surgically induced MI using PET/MRI [56]. Given that GSH-R is also expressed by testicular germ cell tumours, as demonstrated by previously illustrated studies [49–52], the application of this particular ghrelin-analogue PET tracers should be studied in this context. [^18^F]LCE470, being a [^18^F]fluorine-based tracer, has the advantages of high resolution, accessibility, marketability and of low patient radiation burden [57].

### Claudin 6

Tight junctions (TJ) are an essential part of junctional complexes with an important role in cellular polarity, adhesion and permeability, guaranteeing integrity of the tissue structure and with a role in transmembrane movement and signal transduction. Because of this, TJs are one of the first obstacles in tumoral metastatization; indeed, TJs integrity is found decreased in cancerous tissue. TJs are composed of occludin, junction adhesion molecules and claudin (CLDN). The claudin family of protein, counting 27 members, serves a key role regulating barrier functions, differentiation and polarity in epithelial and endothelial cells. The loss of claudins functions is believed to serve an essential role in tumour invasion and metastatization and various studies found that abnormal expression of CLDNs is found in various types of tumours. Claudin 6 (CLDN6) is expressed in numerous cancer histologies but rarely by healthy tissue, making it a possible therapeutic target.

The coding gene of claudin 6 is located on chromosome 16p13.3 and the protein itself weight of 23 kDa. It has 4 transmembrane domains and can bind to signal and cytoskeletal proteins. It is the only member of the CLDNs family to potentially show specificity, as it can activate cell adhesion signals and regulate the activity of nuclear receptors. It is expressed by a wide variety of neoplasms like breast, gastrointestinal (mainly gastric tumours) and genitourinary cancers [58].

CLDN6 role in cancer therapy is widely studied with this protein as target for monoclonal antibodies, antibody-drug conjugates (ADC), bispecific antibodies and CAR-T [59]. In particular, a Chinese preclinical study demonstrated the ability of an ^89^Zr and an ^177^Lu labelled anticlaudin 6 monoclonal antibody ([^89^Zr]Zr-DFO-IMAB027 and [^177^Lu]Lu-DOTA-IMAB027) in imaging and slowing the growth of oesophageal cancer models [60].

Regarding GCTs, expression of claudin 6 in those tumours is well demonstrated since more than 10 years. In 2012, a Japanese study investigated the expression of this protein on the surface of a wide variety of healthy fetal and adult tissues and neoplasms, using immunohistochemistry staining.

Staining was negligible in non-cancerous and in the majority of tumour samples (with the exception for lung large cell carcinoma and some gynaecological cancers) but, regarding TGCTs, a moderate to intense positivity was found in the totality of cases of seminoma, embryonal carcinoma, yolk sac tumour, choriocarcinoma (in the mononucleated trophoblastic component) and focally in the epithelial components of immature teratoma. Unfortunately, no staining was found in spermatocytic seminoma and mature teratoma [61]. Because of the preclinical nature of this evidence and the scarcity of evidence present in literature, further studies are necessary, particularly to determine whether Claudin-6 is commonly expressed in these tumours. A PET tracer based on an antibody or a peptide targeting Claudin-6 could serve as a valuable tool for initial disease staging and for assessing the persistence of viable lesions after treatment. The inability to bind seminocytic seminomas and mature teratomas should be evaluated clinically; especially for the latter, there is a possibility that teratomatous masses resituated after chemotherapy may contain a potentially capturable epithelial component.

Interestingly, some clinical use of claudin 6-based treatments have been already tested. A phase 2 trial evaluated the efficacy of ASP1650, chimeric-mouse/human-IgG1 antibody directed against claudin 6, in a population of 19 men with relapsed or refractory germ cell tumours. Unfortunately, no responses were registered, probably because the simple binding of an antibody may not always be sufficient to induce cell death, and because the immunohistochemical data on expression was evaluated on archived tissue, so the expression could have varied at the time of the study. The most notable result of the study for our purpose is that the immunohistochemistry staining tested positive for CLDN in 94% of patients [62].

A preclinical tested CLDN6-23-ADC, a novel antibody–drug conjugate targeting CLDN6 and carrying monomethyl auristatin E (MMAE), on gynaecological cancer xenografts. The ADC showed selective binding, rapid internalization, and strong antitumor activity with tumour regressions in CLDN6-positive xenografts and it is currently being evaluated in a phase I clinical trial [63].

**Fig. 2:**
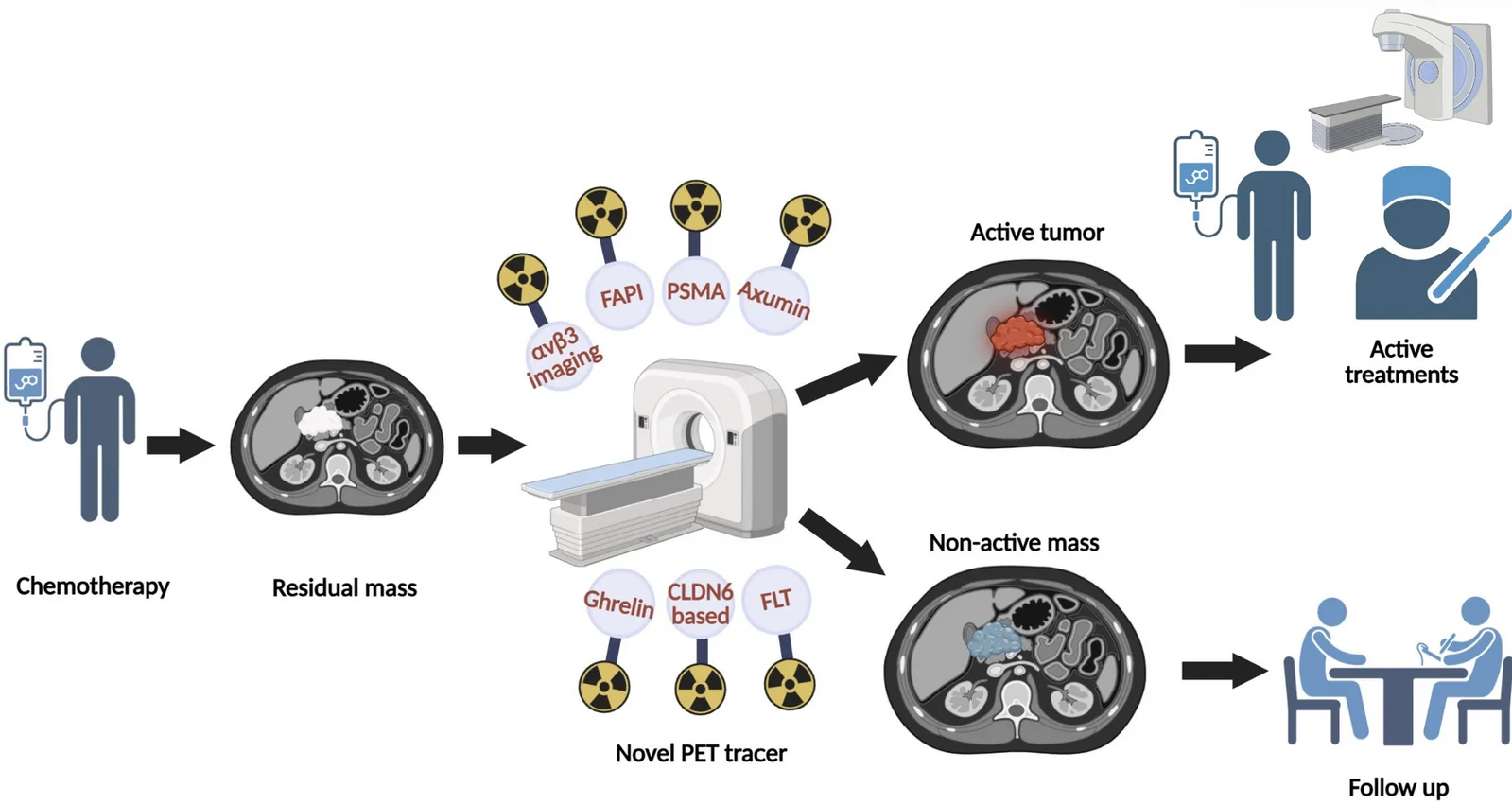
Flowchart of the clinical course of a patient with a residual GCT mass after chemotherapy, assuming the availability of an ideal novel PET tracer. Created with BioRender.com

A phase 1/2 trial, published in 2023 but still ongoing, tested CAR-T cells targeting the CLDN6 with or without a CAR-T cell-amplifying RNA vaccine at two dose levels in relapsed/refractory CLDN6-positive solid tumours. A totality of 22 patients with heavily pretreated relapsed/refractory solid tumours were administered engineered CAR-T, 13 of which men with testicular germ cell tumours. In this subgroup the overall response rate was 38%, which seemed related to the administered dose levels and, moreover, those patients were the ones with the longest duration of response [64]. This is an early clinical study, in a small, heavily pretreated and heterogeneous population but results are promising, especially for TGCTs, and in fact the trial is still going on. Other than the possible further therapeutic alternative for those patients, results of this trial demonstrate that CLDN6 is a viable target and is expressed in TGCTs contributing to providing evidence regarding the potential use of a PET tracer targeting this antigen (Fig. 2).

## Discussion and conclusions

Despite being relatively rare in the overall population, testicular germ cell tumours, is one of the most frequent cancers in young men [65], thus requiring challenging therapies.

PET scans, and nuclear medicine in general, are becoming more fundamental for clinical practice in oncology and [^18^F]-FDG is certainly the most common tracer used in tumour diagnostic, but with important limitations in germinal pathology. Moreover, the opportunity of employing theranostic agents open the view for alternative treatment approaches.

The assessment of residual masses after chemotherapy in GCTs is an open topic of discussion and we understand how the high cases of falsely positive [^18^F]-FDG PET scans in seminomatous residual masses are a common problem encountered in clinical practice.

On the other hand, in advanced non-seminoma a high number of patients with complete response to chemotherapy are treated with unnecessary major surgery. In fact, the surgical resection, with its burden of adverse effects, often revealed necrotic lesions and could be perceived as unnecessary overtreatment. In addition, [^18^F]-FDG PET scans, in this setting, have limited predictive value [12, 66].

The PET scan, given its whole-body coverage, its capability of showing the metabolic activity of a lesion and the possibility of combining an anatomical examination in the same technique (ex. PET/CT scan or PET/MRI) could be successfully exploited to master the staging of this disease and characterization of the nature of the residual lesions.

It is easily knowledgeable that a potential method capable of reliably recognizing viable tumour cells could avoid overtreatment and numerous expensive radiological evaluations, permitting the right choice of treatment for patients.

Furthermore, a method successfully capable of distinguishing the nature of a residual mass after the completion of chemotherapy in metastatic GCTs, could potentially be useful also in early diseases.

These considerations have led to the search for more sensitive and specific PET tracers to optimize GCTs diagnosis, staging and treatment planning. Thus, the growing interest in applying nuclear medicine in clinical practice is also spreading in GCTs. A comparative overview of the main advantages and limitations of the evaluated tracers is provided in Table 2.

**Table 2:** Brief summary of tracers with pros and cons

TRACER	TARGET	STUDY	PROS	CONS
Radioimmunoscintigraphy (anti HCG/AFP ^131^I labelled antybodies, anti PLAP ^111^In–labeled antibody)	HCG/AFP, PLAP	Javadpour et al., 1981 [23] Epenetos et al., 1985 [24]	• Historical proof-of-concept for antigen-targeted imaging • potential tumour-specific uptake	• Limited evidence • Poor spatial resolution • Immunogenicity concerns • Superseded by modern imaging techniques
Thymidine analog 39-deoxy-39-[ ^18^ F]-fluorothymidine (FLT)	Thymidine kinase 1	Pfannenberg et al., 2010 [26]	• Proliferation marker • FLT based tracers available and clinically tested • [^18^F] based tracer (high resolution, accessibility, marketability)	• Demonstrated not to be reliable in diagnosing teratoma
[ ^99 m^ Tc]HYNIC-RGD 6-hydrazinonicotinic acid conjugated to cyclo(Arg-Gly-Asp-D-Phe-Lys)	αvβ3	Aide et al., 2011[38]	• Capable of distinguishing mature teratoma in murine models • Specific for mature teratoma	• No available data from the TERMATEP clinical trial to date
^68^ Ga-PSMA	PSMA	Has Simsek et al., 2021[46]	• Readily available, proven and widespread technology • Possible therapeutic correlation (^177^Lu-PSMA)	• No furher data other than a case report without a full text available • No data about the results of therapy with ^177^Lu-PSMA
[ ^18^ F]-fluciclovine (Axumin)	Sodium-dependent amino acid transporters	TESTPET Woldu et al., 2021[40]	• Readily available and proven technology • [^18^F] based tracer (high resolution, accessibility, marketability)	• Not reliable in diagnosing residual disease or teratoma
[ ^18^ F]-FAPI-74	Fibroblast-activation protein (FAP)	Kidess et al., 2025[39]	• Provides molecular imaging of FAP expression, reflecting tumor microenvironment activity • [^18^F] based tracer (high resolution, accessibility, marketability)	• Very small sample size and pilot design • Tracer uptake can occur in non-viable tissue
[ ^18^ F]LCE470 and other ghrelin-based tracers	Ghrelin receptors (GHS-R1 and GHS-R2)	N.A.	• Great interest in developing ghrelin-based PET tracers • Data about the expression of ghrelin receptors in TGCTs • Existing ghrelin-based tracers • [^18^F] based tracer (high resolution, accessibility, marketability)	• No preclinical or clinical data about the use of ghrelin-based receptors in TGTs • No approved ghrelin-based receptors
Claudin 6 binding tracers	Claudin 6	N.A.	• Known expression of claudin 6 in TGTs • Claudin 6 rarely expressed in healty tissue • Clinical trial currently studying claudin 6 targeting CAR-Ts • Existing ^89^Zr and an ^177^Lu labeled anticlaudin 6 monoclonal antibodies	• No existing claudin 6 based tracers • No preclinical or clinical data about the use of claudin 6 based tracers in TGTs or other tumors

Although PET tracers currently represent the most promising field in molecular imaging of testicular cancer because of their superior spatial resolution and quantitative capabilities, gamma-emitting radiotracers historically contributed to the development of targeted imaging approaches in this disease. Early radioimmunoscintigraphic experiences and more recent SPECT-based tracers targeting angiogenesis-related pathways, such as αvβ3 integrin imaging, demonstrated the feasibility of non-invasive molecular characterization of testicular tumors. However, technical limitations of SPECT imaging, including lower spatial resolution and reduced sensitivity compared with PET/CT, have limited their widespread clinical application. Nevertheless, these approaches retain translational interest, particularly as proof-of-concept experiences for the development of novel PET radiotracers directed against the same biological targets.

In this review we have identified as particularly promising the αvβ3-integrin, the ghrelin-based and anticlaudin 6 based tracers. The first had been already tested in a preclinical scenario with success and an in-vivo study is undergoing. Unfortunately, no results of this trial (TERMATEP) have been yet published to this date, and the preclinical results have not been replicated by other research teams. If the results of this clinical trial will be positive, we would have a readily available, safe and effective tracer capable of distinguishing residual teratomatous masses. There is more data about the expression of ghrelin receptor in tumour tissue, including GCTs and a larger number of ghrelin-based tracers have been developed but none of these have been tested in humans, neither in a clinical scenario. Despite needing a fist in human study to demonstrate the safety and effectiveness in distinguishing vital residual masses in this context, there is robust evidence about the expression of ghrelin receptors in various germinal tumours and because of that the exploitation of a ghrelin-based tracer could provide a very effective tool for the managing of this neoplasm. Claudin 6 have been and is currently being studied as a promising target in TGTs therapy, anti-claudin 6 antibodies are already available and have been proven capable of in vivo binding with their target without excessive tossicity [59]. The aforementioned Chinese preclinical experience [60] highlighted the possibility of developing a labelled anti-claudin 6 antibodies with great potentiality in nuclear imaging but human testing is needed. Therefore, more research is thus needed to fully understand the potential of new agents in this context.

### Possible application in theranostic

Beyond diagnostic imaging, the identification of novel molecular targets and tracers in GCTs may also open future theranostic perspectives. We consider this aspect particularly interesting, since one of the key requirements for an effective diagnostic target is its preferential expression by tumour cells. The combination with an appropriate radionuclide could potentially enable the development of targeted radionuclide therapy, as already established with ^177^Lu-based approaches in prostate cancer [67]. Furthermore, positivity on PET imaging with a specific tracer could potentially identify patients who may benefit from innovative targeted treatments, such as ADCs. We suppose that since αvβ3, FAP, ghrelin-receptors and CLDN6 seem to be expressed preferentially by GCTs, a tracer with a properly chosen radionuclide could be exploited for radionuclide therapy. An emerging body of preclinical studies are evaluating [^177^Lu]Lu-FAPI as a possible agent for radioligand therapy [68], while HYNIC-RGD or LCE470 could be engineered to carry a molecule like ^177^Lu and administered to try to ablate residual disease in GCT patients. The aforementioned Turkish case report of a ^68^Ga-PSMA positive patients treated with ^177^Lu-PSMA [46] is an great example of theranostic in this case, unfortunately additional information about that specific case is not available and there is no evidence from clinical trials of the diagnostic reliability of PSMA PET in GCTs, more research is then needed. Claudin 6 could be another example of theranostic where patients GCT residual masses positive to CLDN6-based receptors could be treated with CLDN6-targeting CAR-T, ADCs or radionuclides.

## Supplementary Information

Below is the link to the electronic supplementary material.

Supplementary Material 1 (PDF 3.94 MB)

## Data Availability

All data analysed during this study are included in this published article and its supplementary information file.
