# Supplementary material for: Novel PET tracers to distinguish the nature of residual masses after the completion of chemotherapy in metastatic testicular germ cell tumours: A systematic review
Source: Eur J Nucl Med Mol Imaging. 2026 Jul 2;53(12):6762–74. doi: 10.1007/s00259-026-08044-5 (PMC13633289; doi:10.1007/s00259-026-08044-5)
Supplement: Supplementary file 1 [file 259_2026_8044_MOESM1_ESM.pdf]

CASP Checklist:
For diagnostic test studies

Reviewer Name:	Alessandro Bertocchi, Laura Evangelista
Paper Title:	The Role of Radioimmunodetection in the Management of Testicular Cancer
Author:	Nasser Javadpour, MD; E. Edmund Kim, MD; Frank H. DeLand, MD<br>Jimmy R. Salyer; Usha Shah; David M. Goldenberg, ScD, MD
Web Link:	https://jamanetwork.com/journals/jama/article-abstract/359050
Appraisal Date:	12/05/2026

During critical appraisal, never make assumptions about what the researchers have done. If it is not possible to tell, use the "Can't tell" response box. If you can't tell, at best it means the researchers have not been explicit or transparent, but at worst it could mean the researchers have not undertaken a particular task or process. Once you've finished the critical appraisal, if there are a large number of "Can't tell" responses, consider whether the findings of the study are trustworthy and interpret the results with caution.

Section A: Are the results of the study valid?	
1. Did the study address a clearly formulated research question?	☑Yes ☐ No ☐ Can't Tell
CONSIDER:<br>*A question should include information about*<br>• *the population*<br>• *the test*<br>• *the setting*<br>• *the outcomes*	
2. Was there a comparison with an appropriate reference standard?	☑Yes ☐ No ☐ Can't Tell
CONSIDER:<br>• *Is this reference test(s) the best available indicator in the circumstances*	
3. Did all patients get the diagnostic test and reference standard?	☑Yes ☐ No ☐ Can't Tell
CONSIDER:<br>• *were both received regardless of the results of the test of interest*<br>• *Check the 2x2 table (verification bias)*	
4. Could the results of the test have been influenced by the results of the reference standard?	☑Yes ☐ No ☐ Can't Tell
CONSIDER:<br>• *was there blinding*<br>• *were the tests performed independently*<br>• *could there have been review bias*	
5. Is the disease status of the tested population clearly described?	☑Yes ☐ No ☐ Can't Tell

CONSIDER:<br>• *presenting symptoms*<br>• *disease stage of severity*<br>• *co-morbidity*<br>• *differential diagnoses (spectrum bias)*	
6. Were the methods for performing the test described in sufficient detail?	☑Yes ☐ No ☐ Can't Tell
CONSIDER:<br>• *Was a protocol followed*	
Section B: What are the results?	
7. What are the results?	☑Yes ☐ No ☐ Can't Tell
CONSIDER:<br>• *are the sensitivity and specificity and/or likelihood ratios presented*<br>• *are the results presented in such a way that we can work them out*	
8. How sure are we about the results? Consequences and cost of alternatives performed?	☑Yes ☐ No ☐ Can't Tell
CONSIDER:<br>• *could they have occurred by chance*<br>• *are there confidence limits*<br>• *what are they*	
Section C: Will the results help locally?	
9. Can the results be applied to your patients/the population of interest?	☑Yes ☐ No ☐ Can't Tell
CONSIDER:<br>• *Do you think your patients/population are so different from those in the study that the results cannot be applied, such as age, sex, ethnicity and spectrum bias.*	

10. Can the test be applied to your patient or population of interest?	☑Yes ☐ No ☐ Can't Tell
CONSIDER:<br>• *resources and opportunity costs*<br>• *level and availability of expertise required to interpret the tests*<br>• *current practice and availability of services*	
11. Were all outcomes important to the individual or population considered?	☑Yes ☐ No ☐ Can't Tell
CONSIDER:<br>• *will the knowledge of the test result improve patient wellbeing*<br>• *will the knowledge of the test result lead to a change in patient management*	
12. What would be the impact of using this test on your patients/population?<br><br>The use of this radioimmunodetection test enhances precision in tumor localization, potentially optimizing pretreatment staging and refining therapeutic selection for patients with testicular cancer	

**APPRAISAL SUMMARY**: *List key points from your critical appraisal that need to be considered when assessing the validity of the results and their usefulness in decision-making.*		
**Positive/Methodologically sound**	**Negative/Relatively poor methodology**	**Unknowns**
The study features a well-defined technical protocol with dual-isotope background subtraction to actively reduce blood-pool artifacts and enhance scan interpretability.	Non-blinded sample size and the complete lack of a systematic gold-standard comparison or statistical analysis.	

Referencing recommendation:

CASP recommends using the Harvard style referencing, which is an author/date method. Sources are cited within the body of your assignment by giving the name of the author(s) followed by the date of publication. All other details about the publication are given in the list of references or bibliography at the end.

Example:

Critical Appraisal Skills Programme (2024). CASP (insert name of checklist i.e. Diagnostic test Checklist.) [online] Available at: insert URL. Accessed: insert date accessed.

Creative Commons

©CASP this work is licensed under the Creative Commons Attribution – Non-Commercial- Share A like. To view a copy of this licence, visit https://creativecommons.org/licenses/by-nc-sa/4.0/

Need further training on evidence-based decision making? Our online training courses are helpful for healthcare educational researchers and any other learners who:

- Need to critically appraise and stay abreast of the healthcare research literature as part of their clinical duties.
- Are considering carrying out research & developing their own research projects.
- Make decisions in their role, whether that be policy making or patient facing.

Benefits of CASP Training:

⇒ Affordable – courses start from as little as £6
⇒ Professional training – leading experts in critical appraisal training
⇒ Self-directed study – complete each course in your own time
⇒ 12 months access – revisit areas you aren't sure of and revise
⇒ CPD certification - after each completed module

Scan the QR code below or visit https://casp-uk.net/critical-appraisal-online-training-courses/ for more information and to start learning more.

CASP Checklist:
For diagnostic test studies

Reviewer Name:	Alessandro Bertocchi, Laura Evangelista
Paper Title:	The Role of Radioimmunodetection in the Management of Testicular Cancer
Author:	Nasser Javadpour, MD; E. Edmund Kim, MD; Frank H. DeLand, MD<br>Jimmy R. Salyer; Usha Shah; David M. Goldenberg, ScD, MD
Web Link:	https://jamanetwork.com/journals/jama/article-abstract/359050
Appraisal Date:	12/05/2026

During critical appraisal, never make assumptions about what the researchers have done. If it is not possible to tell, use the "Can't tell" response box. If you can't tell, at best it means the researchers have not been explicit or transparent, but at worst it could mean the researchers have not undertaken a particular task or process. Once you've finished the critical appraisal, if there are a large number of "Can't tell" responses, consider whether the findings of the study are trustworthy and interpret the results with caution.

Section A: Are the results of the study valid?	
1. Did the study address a clearly formulated research question?	☑Yes ☐ No ☐ Can't Tell
CONSIDER:<br>*A question should include information about*<br>• *the population*<br>• *the test*<br>• *the setting*<br>• *the outcomes*	
2. Was there a comparison with an appropriate reference standard?	☑Yes ☐ No ☐ Can't Tell
CONSIDER:<br>• *Is this reference test(s) the best available indicator in the circumstances*	
3. Did all patients get the diagnostic test and reference standard?	☑Yes ☐ No ☐ Can't Tell
CONSIDER:<br>• *were both received regardless of the results of the test of interest*<br>• *Check the 2x2 table (verification bias)*	
4. Could the results of the test have been influenced by the results of the reference standard?	☑Yes ☐ No ☐ Can't Tell
CONSIDER:<br>• *was there blinding*<br>• *were the tests performed independently*<br>• *could there have been review bias*	
5. Is the disease status of the tested population clearly described?	☑Yes ☐ No ☐ Can't Tell

CONSIDER:<br>• *presenting symptoms*<br>• *disease stage of severity*<br>• *co-morbidity*<br>• *differential diagnoses (spectrum bias)*	
6. Were the methods for performing the test described in sufficient detail?	☑Yes ☐ No ☐ Can't Tell
CONSIDER:<br>• *Was a protocol followed*	
Section B: What are the results?	
7. What are the results?	☑Yes ☐ No ☐ Can't Tell
CONSIDER:<br>• *are the sensitivity and specificity and/or likelihood ratios presented*<br>• *are the results presented in such a way that we can work them out*	
8. How sure are we about the results? Consequences and cost of alternatives performed?	☑Yes ☐ No ☐ Can't Tell
CONSIDER:<br>• *could they have occurred by chance*<br>• *are there confidence limits*<br>• *what are they*	
Section C: Will the results help locally?	
9. Can the results be applied to your patients/the population of interest?	☑Yes ☐ No ☐ Can't Tell
CONSIDER:<br>• *Do you think your patients/population are so different from those in the study that the results cannot be applied, such as age, sex, ethnicity and spectrum bias.*	

10. Can the test be applied to your patient or population of interest?	☑Yes ☐ No ☐ Can't Tell
CONSIDER:<br>• *resources and opportunity costs*<br>• *level and availability of expertise required to interpret the tests*<br>• *current practice and availability of services*	
11. Were all outcomes important to the individual or population considered?	☑Yes ☐ No ☐ Can't Tell
CONSIDER:<br>• *will the knowledge of the test result improve patient wellbeing*<br>• *will the knowledge of the test result lead to a change in patient management*	
12. What would be the impact of using this test on your patients/population?<br><br>The use of this radioimmunodetection test enhances precision in tumor localization, potentially optimizing pretreatment staging and refining therapeutic selection for patients with testicular cancer	

**APPRAISAL SUMMARY**: *List key points from your critical appraisal that need to be considered when assessing the validity of the results and their usefulness in decision-making.*		
**Positive/Methodologically sound**	**Negative/Relatively poor methodology**	**Unknowns**
The study features a well-defined technical protocol with dual-isotope background subtraction to actively reduce blood-pool artifacts and enhance scan interpretability.	Non-blinded sample size and the complete lack of a systematic gold-standard comparison or statistical analysis.	

Referencing recommendation:

CASP recommends using the Harvard style referencing, which is an author/date method. Sources are cited within the body of your assignment by giving the name of the author(s) followed by the date of publication. All other details about the publication are given in the list of references or bibliography at the end.

Example:

Critical Appraisal Skills Programme (2024). CASP (insert name of checklist i.e. Diagnostic test Checklist.) [online] Available at: insert URL. Accessed: insert date accessed.

Creative Commons

©CASP this work is licensed under the Creative Commons Attribution – Non-Commercial- Share A like. To view a copy of this licence, visit https://creativecommons.org/licenses/by-nc-sa/4.0/

Need further training on evidence-based decision making? Our online training courses are helpful for healthcare educational researchers and any other learners who:

- Need to critically appraise and stay abreast of the healthcare research literature as part of their clinical duties.
- Are considering carrying out research & developing their own research projects.
- Make decisions in their role, whether that be policy making or patient facing.

Benefits of CASP Training:

⇒ Affordable – courses start from as little as £6
⇒ Professional training – leading experts in critical appraisal training
⇒ Self-directed study – complete each course in your own time
⇒ 12 months access – revisit areas you aren't sure of and revise
⇒ CPD certification - after each completed module

Scan the QR code below or visit https://casp-appraisal-online-uk.net/critical-training-courses/ for more information and to start learning more.

CASP Checklist:
For Qualitative Research

Reviewer Name:	Alessandro Bertocchi, Laura Evangelista
Paper Title:	αvβ3 imaging can accurately distinguish between mature teratoma and necrosis in 18F-FDG-negative residual masses after treatment of non-seminomatous testicular cancer: a preclinical study
Author:	Nicolas Aide, Mélanie Briand, Pierre Bohn, Soizic Dutoit, Charline Lasnon, Jacques Chasle, Jean Rouvet, Romain Modzelewski, Antony Vela, Edwiges Deslandes, Pierre Vera, Laurent Poulain, Franck Carreiras
Web Link:	https://link-springer-com.humanitas.idm.oclc.org/article/10.1007/s00259-010-1624-9
Appraisal Date:	01/09/2025

During critical appraisal, never make assumptions about what the researchers have done. If it is not possible to tell, use the "Can't tell" response box. If you can't tell, at best it means the researchers have not been explicit or transparent, but at worst it could mean the researchers have not undertaken a particular task or process. Once you've finished the critical appraisal, if there are a large number of "Can't tell" responses, consider whether the findings of the study are trustworthy and interpret the results with caution.

Section A Are the results valid?	
1. Was there a clear statement of the aims of the research?	☒ Yes ☐ No ☐ Can't Tell
CONSIDER:<br>• *what was the goal of the research?*<br>• *why was it thought important?*<br>• *its relevance*	
2. Is a qualitative methodology appropriate?	☒ Yes ☐ No ☐ Can't Tell
CONSIDER:<br>• *If the research seeks to interpret or illuminate the actions and/or subjective experiences of research participants*<br>• *Is qualitative research the right methodology for addressing the research goal?*	
3. Was the research design appropriate to address the aims of the research?	☒ Yes ☐ No ☐ Can't Tell
CONSIDER:<br>• *if the researcher has justified the research design (e.g., have they discussed how they decided which method to use)*	
4. Was the recruitment strategy appropriate to the aims of the research?	☒ Yes ☐ No ☐ Can't Tell
CONSIDER:<br>• *If the researcher has explained how the participants were selected*<br>• *If they explained why the participants they selected were the most appropriate to provide access to the type of knowledge sought by the study*<br>• *If there are any discussions around recruitment (e.g. why some people chose not to take part)*	

5. Was the data collected in a way that addressed the research issue?	☒ Yes ☐ No ☐ Can't Tell

CONSIDER:

- *If the setting for the data collection was justified*
- *If it is clear how data were collected (e.g. focus group, semi-structured interview etc.)*
- *If the researcher has justified the methods chosen*
- *If the researcher has made the methods explicit (e.g. for interview method, is there an indication of how interviews are conducted, or did they use a topic guide)*
- *If methods were modified during the study. If so, has the researcher explained how and why*
- *If the form of data is clear (e.g. tape recordings, video material, notes etc.)*
- *If the researcher has discussed saturation of data*

6. Has the relationship between researcher and participants been adequately considered?	☒ Yes ☐ No ☐ Can't Tell

CONSIDER:

- *If the researcher critically examined their own role, potential bias and influence during (a) formulation of the research questions (b) data collection, including sample recruitment and choice of location*
- *How the researcher responded to events during the study and whether they considered the implications of any changes in the research design*

Section B: What are the results?

7. Have ethical issues been taken into consideration?	☒ Yes ☐ No ☐ Can't Tell

CONSIDER:

- *If there are sufficient details of how the research was explained to participants for the reader to assess whether ethical standards were maintained*
- *If the researcher has discussed issues raised by the study (e.g. issues around informed consent or confidentiality or how they have handled the effects of the study on the participants during and after the study)*
- *If approval has been sought from the ethics committee*

8. Was the data analysis sufficiently rigorous?	☒ Yes ☐ No ☐ Can't Tell
CONSIDER:<br>• *If there is an in-depth description of the analysis process*<br>• *If thematic analysis is used. If so, is it clear how the categories/themes were derived from the data*<br>• *Whether the researcher explains how the data presented were selected from the original sample to demonstrate the analysis process*<br>• *If sufficient data are presented to support the findings*<br>• *To what extent contradictory data are taken into account*<br>• *Whether the researcher critically examined their own role, potential bias and influence during analysis and selection of data for presentation*	
9. Is there a clear statement of findings?	☒ Yes ☐ No ☐ Can't Tell
CONSIDER:<br>• *If the findings are explicit*<br>• *If there is adequate discussion of the evidence both for and against the researcher's arguments*<br>• *If the researcher has discussed the credibility of their findings (e.g. triangulation, respondent validation, more than one analyst)*<br>• *If the findings are discussed in relation to the original research question*	
Section C: Will the results help locally?	
10. How valuable is the research?	☒ Yes ☐ No ☐ Can't Tell
CONSIDER:<br>• *If the researcher discusses the contribution the study makes to existing knowledge or understanding (e.g., do they consider the findings in relation to current practice or policy, or relevant research-based literature*<br>• *If they identify new areas where research is necessary*<br>• *If the researchers have discussed whether or how the findings can be transferred to other populations or considered other ways the research may be used*	

**APPRAISAL SUMMARY**: *List key points from your critical appraisal that need to be considered when assessing the validity of the results and their usefulness in decision-making.*		
**Positive/Methodologically sound**	**Negative/Relatively poor methodology**	**Unknowns**
Division of models between mature teratoma and necrosis	Small number of subjects	

Referencing recommendation:

CASP recommends using the Harvard style referencing, which is an author/date method. Sources are cited within the body of your assignment by giving the name of the author(s) followed by the date of publication. All other details about the publication are given in the list of references or bibliography at the end.

Example:

Critical Appraisal Skills Programme (2024). CASP (insert name of checklist i.e. systematic reviews with meta-analysis of randomised controlled trials (RCTs) Checklist.) [online] Available at: insert URL. Accessed: insert date accessed.

Creative Commons

©CASP this work is licensed under the Creative Commons Attribution – Non-Commercial- Share A like. To view a copy of this licence, visit https://creativecommons.org/licenses/by-nc-sa/4.0/

Need further training on evidence-based decision making? Our online training courses are helpful for healthcare educational researchers and any other learners who:

- Need to critically appraise and stay abreast of the healthcare research literature as part of their clinical duties.
- Are considering carrying out research & developing their own research projects.
- Make decisions in their role, whether that be policy making or patient facing.

Benefits of CASP Training:

⇒ Affordable – courses start from as little as £6
⇒ Professional training – leading experts in critical appraisal training
⇒ Self-directed study – complete each course in your own time
⇒ 12 months access – revisit areas you aren't sure of and revise
⇒ CPD certification - after each completed module

Scan the QR code below or visit https://casp-uk.net/critical-appraisal-online-training-courses/ for more information and to start learning more.

CASP Checklist:
For Qualitative Research

Reviewer Name:	Alessandro Bertocchi, Laura Evangelista
Paper Title:	PET/CT with 18F-FLT: Does It Improve the Therapeutic Management of Metastatic Germ Cell Tumors?
Author:	Christina Pfannenberg, Philip Aschoff, Helmut Dittmann, Frank Mayer, Gerald Reischl Claus von Weyhern, Lothar Kanz2 Claus D. Claussen, Roland Bares, and Joerg T. Hartmann
Web Link:	https://jnm.snmjournals.org/content/51/6/845.long
Appraisal Date:	01/09/2025

During critical appraisal, never make assumptions about what the researchers have done. If it is not possible to tell, use the "Can't tell" response box. If you can't tell, at best it means the researchers have not been explicit or transparent, but at worst it could mean the researchers have not undertaken a particular task or process. Once you've finished the critical appraisal, if there are a large number of "Can't tell" responses, consider whether the findings of the study are trustworthy and interpret the results with caution.

Section A Are the results valid?	
1. Was there a clear statement of the aims of the research?	☒Yes ☐ No ☐ Can't Tell
CONSIDER:<br>• *what was the goal of the research?*<br>• *why was it thought important?*<br>• *its relevance*	
2. Is a qualitative methodology appropriate?	☒Yes ☐ No ☐ Can't Tell
CONSIDER:<br>• *If the research seeks to interpret or illuminate the actions and/or subjective experiences of research participants*<br>• *Is qualitative research the right methodology for addressing the research goal?*	
3. Was the research design appropriate to address the aims of the research?	☒Yes ☐ No ☐ Can't Tell
CONSIDER:<br>• *if the researcher has justified the research design (e.g., have they discussed how they decided which method to use)*	
4. Was the recruitment strategy appropriate to the aims of the research?	☒Yes ☐ No ☐ Can't Tell
CONSIDER:<br>• *If the researcher has explained how the participants were selected*<br>• *If they explained why the participants they selected were the most appropriate to provide access to the type of knowledge sought by the study*<br>• *If there are any discussions around recruitment (e.g. why some people chose not to take part)*	

5. Was the data collected in a way that addressed the research issue?	☒ Yes ☐ No ☐ Can't Tell
CONSIDER:<br>• *If the setting for the data collection was justified*<br>• *If it is clear how data were collected (e.g. focus group, semi-structured interview etc.)*<br>• *If the researcher has justified the methods chosen*<br>• *If the researcher has made the methods explicit (e.g. for interview method, is there an indication of how interviews are conducted, or did they use a topic guide)*<br>• *If methods were modified during the study. If so, has the researcher explained how and why*<br>• *If the form of data is clear (e.g. tape recordings, video material, notes etc.)*<br>• *If the researcher has discussed saturation of data*	
6. Has the relationship between researcher and participants been adequately considered?	☐ Yes ☒ No ☐ Can't Tell
CONSIDER:<br>• *If the researcher critically examined their own role, potential bias and influence during (a) formulation of the research questions (b) data collection, including sample recruitment and choice of location*<br>• *How the researcher responded to events during the study and whether they considered the implications of any changes in the research design*	
**Section B: What are the results?**	
7. Have ethical issues been taken into consideration?	☐ Yes ☒ No ☐ Can't Tell
CONSIDER:<br>• *If there are sufficient details of how the research was explained to participants for the reader to assess whether ethical standards were maintained*<br>• *If the researcher has discussed issues raised by the study (e.g. issues around informed consent or confidentiality or how they have handled the effects of the study on the participants during and after the study)*<br>• *If approval has been sought from the ethics committee*	

8. Was the data analysis sufficiently rigorous?	☒ Yes ☐ No ☐ Can't Tell
CONSIDER:<br>• *If there is an in-depth description of the analysis process*<br>• *If thematic analysis is used. If so, is it clear how the categories/themes were derived from the data*<br>• *Whether the researcher explains how the data presented were selected from the original sample to demonstrate the analysis process*<br>• *If sufficient data are presented to support the findings*<br>• *To what extent contradictory data are taken into account*<br>• *Whether the researcher critically examined their own role, potential bias and influence during analysis and selection of data for presentation*	
9. Is there a clear statement of findings?	☒ Yes ☐ No ☐ Can't Tell
CONSIDER:<br>• *If the findings are explicit*<br>• *If there is adequate discussion of the evidence both for and against the researcher's arguments*<br>• *If the researcher has discussed the credibility of their findings (e.g. triangulation, respondent validation, more than one analyst)*<br>• *If the findings are discussed in relation to the original research question*	
Section C: Will the results help locally?	
10. How valuable is the research?	☒ Yes ☐ No ☐ Can't Tell
CONSIDER:<br>• *If the researcher discusses the contribution the study makes to existing knowledge or understanding (e.g., do they consider the findings in relation to current practice or policy, or relevant research-based literature*<br>• *If they identify new areas where research is necessary*<br>• *If the researchers have discussed whether or how the findings can be transferred to other populations or considered other ways the research may be used*	

**APPRAISAL SUMMARY**: *List key points from your critical appraisal that need to be considered when assessing the validity of the results and their usefulness in decision-making.*		
**Positive/Methodologically sound**	**Negative/Relatively poor methodology**	**Unknowns**
Clear description of methods<br><br>Clear description of the results, even if negative	No consideration about ethical implications	

Referencing recommendation:

CASP recommends using the Harvard style referencing, which is an author/date method. Sources are cited within the body of your assignment by giving the name of the author(s) followed by the date of publication. All other details about the publication are given in the list of references or bibliography at the end.

Example:

Critical Appraisal Skills Programme (2024). CASP (insert name of checklist i.e. systematic reviews with meta-analysis of randomised controlled trials (RCTs) Checklist.) [online] Available at: insert URL. Accessed: insert date accessed.

Creative Commons

©CASP this work is licensed under the Creative Commons Attribution – Non-Commercial- Share A like. To view a copy of this licence, visit https://creativecommons.org/licenses/by-nc-sa/4.0/

Need further training on evidence-based decision making? Our online training courses are helpful for healthcare educational researchers and any other learners who:

- Need to critically appraise and stay abreast of the healthcare research literature as part of their clinical duties.
- Are considering carrying out research & developing their own research projects.
- Make decisions in their role, whether that be policy making or patient facing.

Benefits of CASP Training:

⇒ Affordable – courses start from as little as £6
⇒ Professional training – leading experts in critical appraisal training
⇒ Self-directed study – complete each course in your own time
⇒ 12 months access – revisit areas you aren't sure of and revise
⇒ CPD certification - after each completed module

Scan the QR code below or visit https://casp-uk.net/critical-appraisal-online-training-courses/ for more information and to start learning more.

Critical Appraisal Skills Programme

CASP Checklist:
For Qualitative Research

Reviewer Name:	Alessandro Bertocchi
Paper Title:	177Lu-PSMA Therapy for Metastatic Testicular Mixed Germ Cell Tumor
Author:	Duygu Has Simsek, Caner Civan, Meltem Ekenel, Serkan Kuyumcu, Yasemin Sanli
Web Link:	https://pubmed-ncbi-nlm-nih-gov.humanitas.idm.oclc.org/33630811/
Appraisal Date:	01/09/2025

During critical appraisal, never make assumptions about what the researchers have done. If it is not possible to tell, use the "Can't tell" response box. If you can't tell, at best it means the researchers have not been explicit or transparent, but at worst it could mean the researchers have not undertaken a particular task or process. Once you've finished the critical appraisal, if there are a large number of "Can't tell" responses, consider whether the findings of the study are trustworthy and interpret the results with caution.

Section A Are the results valid?	
1. Was there a clear statement of the aims of the research?	☒ Yes ☐ No ☐ Can't Tell
CONSIDER:<br>• *what was the goal of the research?*<br>• *why was it thought important?*<br>• *its relevance*	
2. Is a qualitative methodology appropriate?	☐ Yes ☒ No ☐ Can't Tell
CONSIDER:<br>• *If the research seeks to interpret or illuminate the actions and/or subjective experiences of research participants*<br>• *Is qualitative research the right methodology for addressing the research goal?*	
3. Was the research design appropriate to address the aims of the research?	☒ Yes ☐ No ☐ Can't Tell
CONSIDER:<br>• *if the researcher has justified the research design (e.g., have they discussed how they decided which method to use)*	
4. Was the recruitment strategy appropriate to the aims of the research?	☐ Yes ☒ No ☐ Can't Tell
CONSIDER:<br>• *If the researcher has explained how the participants were selected*<br>• *If they explained why the participants they selected were the most appropriate to provide access to the type of knowledge sought by the study*<br>• *If there are any discussions around recruitment (e.g. why some people chose not to take part)*	

5. Was the data collected in a way that addressed the research issue?	☐ Yes ☒ No ☐ Can't Tell
CONSIDER:<br>• *If the setting for the data collection was justified*<br>• *If it is clear how data were collected (e.g. focus group, semi-structured interview etc.)*<br>• *If the researcher has justified the methods chosen*<br>• *If the researcher has made the methods explicit (e.g. for interview method, is there an indication of how interviews are conducted, or did they use a topic guide)*<br>• *If methods were modified during the study. If so, has the researcher explained how and why*<br>• *If the form of data is clear (e.g. tape recordings, video material, notes etc.)*<br>• *If the researcher has discussed saturation of data*	
6. Has the relationship between researcher and participants been adequately considered?	☐ Yes ☒ No ☐ Can't Tell
CONSIDER:<br>• *If the researcher critically examined their own role, potential bias and influence during (a) formulation of the research questions (b) data collection, including sample recruitment and choice of location*<br>• *How the researcher responded to events during the study and whether they considered the implications of any changes in the research design*	
**Section B: What are the results?**	
7. Have ethical issues been taken into consideration?	☐ Yes ☒ No ☐ Can't Tell
CONSIDER:<br>• *If there are sufficient details of how the research was explained to participants for the reader to assess whether ethical standards were maintained*<br>• *If the researcher has discussed issues raised by the study (e.g. issues around informed consent or confidentiality or how they have handled the effects of the study on the participants during and after the study)*<br>• *If approval has been sought from the ethics committee*	

8. Was the data analysis sufficiently rigorous?	☐ Yes ☒ No ☐ Can't Tell

CONSIDER:
- *If there is an in-depth description of the analysis process*
- *If thematic analysis is used. If so, is it clear how the categories/themes were derived from the data*
- *Whether the researcher explains how the data presented were selected from the original sample to demonstrate the analysis process*
- *If sufficient data are presented to support the findings*
- *To what extent contradictory data are taken into account*
- *Whether the researcher critically examined their own role, potential bias and influence during analysis and selection of data for presentation*

9. Is there a clear statement of findings?	☒ Yes ☐ No ☐ Can't Tell

CONSIDER:
- *If the findings are explicit*
- *If there is adequate discussion of the evidence both for and against the researcher's arguments*
- *If the researcher has discussed the credibility of their findings (e.g. triangulation, respondent validation, more than one analyst)*
- *If the findings are discussed in relation to the original research question*

Section C: Will the results help locally?

10. How valuable is the research?	☒ Yes ☐ No ☐ Can't Tell

CONSIDER:
- *If the researcher discusses the contribution the study makes to existing knowledge or understanding (e.g., do they consider the findings in relation to current practice or policy, or relevant research-based literature*
- *If they identify new areas where research is necessary*
- *If the researchers have discussed whether or how the findings can be transferred to other populations or considered other ways the research may be used*

**APPRAISAL SUMMARY**: *List key points from your critical appraisal that need to be considered when assessing the validity of the results and their usefulness in decision-making.*		
**Positive/Methodologically sound**	**Negative/Relatively poor methodology**	**Unknowns**
Ga-PSMA PET/CT taken before and after treatment<br><br>Alfafetoprotein measured before and after treatment	Single case report<br><br>No information about the anamnesis of the patient<br><br>No histological correlation<br><br>No preclinical data available	

Referencing recommendation:

CASP recommends using the Harvard style referencing, which is an author/date method. Sources are cited within the body of your assignment by giving the name of the author(s) followed by the date of publication. All other details about the publication are given in the list of references or bibliography at the end.

Example:

Critical Appraisal Skills Programme (2024). CASP (insert name of checklist i.e. systematic reviews with meta-analysis of randomised controlled trials (RCTs) Checklist.) [online] Available at: insert URL. Accessed: insert date accessed.

Creative Commons

©CASP this work is licensed under the Creative Commons Attribution – Non-Commercial- Share A like. To view a copy of this licence, visit https://creativecommons.org/licenses/by-nc-sa/4.0/

Need further training on evidence-based decision making? Our online training courses are helpful for healthcare educational researchers and any other learners who:

- Need to critically appraise and stay abreast of the healthcare research literature as part of their clinical duties.
- Are considering carrying out research & developing their own research projects.
- Make decisions in their role, whether that be policy making or patient facing.

Benefits of CASP Training:

⇒ Affordable – courses start from as little as £6
⇒ Professional training – leading experts in critical appraisal training
⇒ Self-directed study – complete each course in your own time
⇒ 12 months access – revisit areas you aren't sure of and revise
⇒ CPD certification - after each completed module

Scan the QR code below or visit https://casp-uk.net/critical-appraisal-online-training-courses/ for more information and to start learning more.

CASP Checklist:
For Qualitative Research

Reviewer Name:	
Paper Title:	
Author:	
Web Link:	
Appraisal Date:	

During critical appraisal, never make assumptions about what the researchers have done. If it is not possible to tell, use the “Can’t tell” response box. If you can’t tell, at best it means the researchers have not been explicit or transparent, but at worst it could mean the researchers have not undertaken a particular task or process. Once you’ve finished the critical appraisal, if there are a large number of “Can’t tell” responses, consider whether the findings of the study are trustworthy and interpret the results with caution.

Section A Are the results valid?	
1. Was there a clear statement of the aims of the research?	☐ Yes ☐ No ☐ Can't Tell
CONSIDER:<br>• *what was the goal of the research?*<br>• *why was it thought important?*<br>• *its relevance*	
2. Is a qualitative methodology appropriate?	☐ Yes ☐ No ☐ Can't Tell
CONSIDER:<br>• *If the research seeks to interpret or illuminate the actions and/or subjective experiences of research participants*<br>• *Is qualitative research the right methodology for addressing the research goal?*	
3. Was the research design appropriate to address the aims of the research?	☐ Yes ☐ No ☐ Can't Tell
CONSIDER:<br>• *if the researcher has justified the research design (e.g., have they discussed how they decided which method to use)*	
4. Was the recruitment strategy appropriate to the aims of the research?	☐ Yes ☐ No ☐ Can't Tell
CONSIDER:<br>• *If the researcher has explained how the participants were selected*<br>• *If they explained why the participants they selected were the most appropriate to provide access to the type of knowledge sought by the study*<br>• *If there are any discussions around recruitment (e.g. why some people chose not to take part)*	

5. Was the data collected in a way that addressed the research issue?	☐Yes ☐ No ☐ Can't Tell

CONSIDER:

- *If the setting for the data collection was justified*
- *If it is clear how data were collected (e.g. focus group, semi-structured interview etc.)*
- *If the researcher has justified the methods chosen*
- *If the researcher has made the methods explicit (e.g. for interview method, is there an indication of how interviews are conducted, or did they use a topic guide)*
- *If methods were modified during the study. If so, has the researcher explained how and why*
- *If the form of data is clear (e.g. tape recordings, video material, notes etc.)*
- *If the researcher has discussed saturation of data*

6. Has the relationship between researcher and participants been adequately considered?	☐Yes ☐ No ☐ Can't Tell

CONSIDER:

- *If the researcher critically examined their own role, potential bias and influence during (a) formulation of the research questions (b) data collection, including sample recruitment and choice of location*
- *How the researcher responded to events during the study and whether they considered the implications of any changes in the research design*

Section B: What are the results?

7. Have ethical issues been taken into consideration?	☐Yes ☐ No ☐ Can't Tell

CONSIDER:

- *If there are sufficient details of how the research was explained to participants for the reader to assess whether ethical standards were maintained*
- *If the researcher has discussed issues raised by the study (e.g. issues around informed consent or confidentiality or how they have handled the effects of the study on the participants during and after the study)*
- *If approval has been sought from the ethics committee*

8. Was the data analysis sufficiently rigorous?	☐ Yes ☐ No ☐ Can't Tell
CONSIDER:<br>• *If there is an in-depth description of the analysis process*<br>• *If thematic analysis is used. If so, is it clear how the categories/themes were derived from the data*<br>• *Whether the researcher explains how the data presented were selected from the original sample to demonstrate the analysis process*<br>• *If sufficient data are presented to support the findings*<br>• *To what extent contradictory data are taken into account*<br>• *Whether the researcher critically examined their own role, potential bias and influence during analysis and selection of data for presentation*	
9. Is there a clear statement of findings?	☐ Yes ☐ No ☐ Can't Tell
CONSIDER:<br>• *If the findings are explicit*<br>• *If there is adequate discussion of the evidence both for and against the researcher's arguments*<br>• *If the researcher has discussed the credibility of their findings (e.g. triangulation, respondent validation, more than one analyst)*<br>• *If the findings are discussed in relation to the original research question*	
Section C: Will the results help locally?	
10. How valuable is the research?	☐ Yes ☐ No ☐ Can't Tell
CONSIDER:<br>• *If the researcher discusses the contribution the study makes to existing knowledge or understanding (e.g., do they consider the findings in relation to current practice or policy, or relevant research-based literature*<br>• *If they identify new areas where research is necessary*<br>• *If the researchers have discussed whether or how the findings can be transferred to other populations or considered other ways the research may be used*	

**APPRAISAL SUMMARY**: *List key points from your critical appraisal that need to be considered when assessing the validity of the results and their usefulness in decision-making.*		
**Positive/Methodologically sound**	**Negative/Relatively poor methodology**	**Unknowns**

Referencing recommendation:

CASP recommends using the Harvard style referencing, which is an author/date method. Sources are cited within the body of your assignment by giving the name of the author(s) followed by the date of publication. All other details about the publication are given in the list of references or bibliography at the end.

Example:

Critical Appraisal Skills Programme (2024). CASP (insert name of checklist i.e. systematic reviews with meta-analysis of randomised controlled trials (RCTs) Checklist.) [online] Available at: insert URL. Accessed: insert date accessed.

Creative Commons

©CASP this work is licensed under the Creative Commons Attribution – Non-Commercial- Share A like. To view a copy of this licence, visit https://creativecommons.org/licenses/by-nc-sa/4.0/

Need further training on evidence-based decision making? Our online training courses are helpful for healthcare educational researchers and any other learners who:

- Need to critically appraise and stay abreast of the healthcare research literature as part of their clinical duties.
- Are considering carrying out research & developing their own research projects.
- Make decisions in their role, whether that be policy making or patient facing.

Benefits of CASP Training:

⇒ Affordable – courses start from as little as £6
⇒ Professional training – leading experts in critical appraisal training
⇒ Self-directed study – complete each course in your own time
⇒ 12 months access – revisit areas you aren't sure of and revise
⇒ CPD certification - after each completed module

Scan the QR code below or visit https://casp-uk.net/critical-appraisal-online-training-courses/ for more information and to start learning more.

CASP Checklist:
For Qualitative Research

Reviewer Name:	Alessandro Bertocchi, Laura Evangelista
Paper Title:	Performance characteristics of 18F-fluciclovine positron emission tomography/computed tomography prior to retroperitoneal lymph node dissection
Author:	Solomon L Woldu, Xiaosong Meng, Daniel Wong, Fady Baky, Vitaly Margulis, Yin Xi, Rathan M Subramaniam, Aditya Bagrodia
Web Link:	https://cuaj.ca/index.php/journal/article/view/7317
Appraisal Date:	01/09/2025

During critical appraisal, never make assumptions about what the researchers have done. If it is not possible to tell, use the "Can't tell" response box. If you can't tell, at best it means the researchers have not been explicit or transparent, but at worst it could mean the researchers have not undertaken a particular task or process. Once you've finished the critical appraisal, if there are a large number of "Can't tell" responses, consider whether the findings of the study are trustworthy and interpret the results with caution.

Section A Are the results valid?	
1. Was there a clear statement of the aims of the research?	☒ Yes ☐ No ☐ Can't Tell
CONSIDER:<br>• *what was the goal of the research?*<br>• *why was it thought important?*<br>• *its relevance*	
2. Is a qualitative methodology appropriate?	☒ Yes ☐ No ☐ Can't Tell
CONSIDER:<br>• *If the research seeks to interpret or illuminate the actions and/or subjective experiences of research participants*<br>• *Is qualitative research the right methodology for addressing the research goal?*	
3. Was the research design appropriate to address the aims of the research?	☒ Yes ☐ No ☐ Can't Tell
CONSIDER:<br>• *if the researcher has justified the research design (e.g., have they discussed how they decided which method to use)*	
4. Was the recruitment strategy appropriate to the aims of the research?	☒ Yes ☐ No ☐ Can't Tell
CONSIDER:<br>• *If the researcher has explained how the participants were selected*<br>• *If they explained why the participants they selected were the most appropriate to provide access to the type of knowledge sought by the study*<br>• *If there are any discussions around recruitment (e.g. why some people chose not to take part)*	

5. Was the data collected in a way that addressed the research issue?	☒ Yes ☐ No ☐ Can't Tell
CONSIDER:<br>• *If the setting for the data collection was justified*<br>• *If it is clear how data were collected (e.g. focus group, semi-structured interview etc.)*<br>• *If the researcher has justified the methods chosen*<br>• *If the researcher has made the methods explicit (e.g. for interview method, is there an indication of how interviews are conducted, or did they use a topic guide)*<br>• *If methods were modified during the study. If so, has the researcher explained how and why*<br>• *If the form of data is clear (e.g. tape recordings, video material, notes etc.)*<br>• *If the researcher has discussed saturation of data*	
6. Has the relationship between researcher and participants been adequately considered?	☐ Yes ☒ No ☐ Can't Tell
CONSIDER:<br>• *If the researcher critically examined their own role, potential bias and influence during (a) formulation of the research questions (b) data collection, including sample recruitment and choice of location*<br>• *How the researcher responded to events during the study and whether they considered the implications of any changes in the research design*	
Section B: What are the results?	
7. Have ethical issues been taken into consideration?	☒ Yes ☐ No ☐ Can't Tell
CONSIDER:<br>• *If there are sufficient details of how the research was explained to participants for the reader to assess whether ethical standards were maintained*<br>• *If the researcher has discussed issues raised by the study (e.g. issues around informed consent or confidentiality or how they have handled the effects of the study on the participants during and after the study)*<br>• *If approval has been sought from the ethics committee*	

8. Was the data analysis sufficiently rigorous?	☒ Yes ☐ No ☐ Can't Tell

CONSIDER:

- *If there is an in-depth description of the analysis process*
- *If thematic analysis is used. If so, is it clear how the categories/themes were derived from the data*
- *Whether the researcher explains how the data presented were selected from the original sample to demonstrate the analysis process*
- *If sufficient data are presented to support the findings*
- *To what extent contradictory data are taken into account*
- *Whether the researcher critically examined their own role, potential bias and influence during analysis and selection of data for presentation*

9. Is there a clear statement of findings?	☒ Yes ☐ No ☐ Can't Tell

CONSIDER:

- *If the findings are explicit*
- *If there is adequate discussion of the evidence both for and against the researcher's arguments*
- *If the researcher has discussed the credibility of their findings (e.g. triangulation, respondent validation, more than one analyst)*
- *If the findings are discussed in relation to the original research question*

Section C: Will the results help locally?

10. How valuable is the research?	☒ Yes ☐ No ☐ Can't Tell

CONSIDER:

- *If the researcher discusses the contribution the study makes to existing knowledge or understanding (e.g., do they consider the findings in relation to current practice or policy, or relevant research-based literature*
- *If they identify new areas where research is necessary*
- *If the researchers have discussed whether or how the findings can be transferred to other populations or considered other ways the research may be used*

**APPRAISAL SUMMARY**: *List key points from your critical appraisal that need to be considered when assessing the validity of the results and their usefulness in decision-making.*		
**Positive/Methodologically sound**	**Negative/Relatively poor methodology**	**Unknowns**
Correlation between PET/CT and histology	Small number of patients	

Referencing recommendation:

CASP recommends using the Harvard style referencing, which is an author/date method. Sources are cited within the body of your assignment by giving the name of the author(s) followed by the date of publication. All other details about the publication are given in the list of references or bibliography at the end.

Example:

Critical Appraisal Skills Programme (2024). CASP (insert name of checklist i.e. systematic reviews with meta-analysis of randomised controlled trials (RCTs) Checklist.) [online] Available at: insert URL. Accessed: insert date accessed.

Creative Commons

©CASP this work is licensed under the Creative Commons Attribution – Non-Commercial- Share A like. To view a copy of this licence, visit https://creativecommons.org/licenses/by-nc-sa/4.0/

Need further training on evidence-based decision making? Our online training courses are helpful for healthcare educational researchers and any other learners who:

- Need to critically appraise and stay abreast of the healthcare research literature as part of their clinical duties.
- Are considering carrying out research & developing their own research projects.
- Make decisions in their role, whether that be policy making or patient facing.

Benefits of CASP Training:

⇒ Affordable – courses start from as little as £6
⇒ Professional training – leading experts in critical appraisal training
⇒ Self-directed study – complete each course in your own time
⇒ 12 months access – revisit areas you aren't sure of and revise
⇒ CPD certification - after each completed module

Scan the QR code below or visit https://casp-uk.net/critical-appraisal-online-training-courses/ for more information and to start learning more.
